# R/BHC: fast Bayesian hierarchical clustering for microarray data

**DOI:** 10.1186/1471-2105-10-242

**Published:** 2009-08-06

**Authors:** Richard S Savage, Katherine Heller, Yang Xu, Zoubin Ghahramani, William M Truman, Murray Grant, Katherine J Denby, David L Wild

**Affiliations:** 1 Systems Biology Centre, University of Warwick, Coventry House, Coventry, CV4 7AL, UK; 2 Warwick HRI, University of Warwick, Wellesbourne, CV35 9EF, UK; 3 Department of Engineering, University of Cambridge, Cambridge CB2 1PZ, UK; 4 School of Biosciences, University of Exeter, Exeter, EX4 4QD, UK

## Abstract

**Background:**

Although the use of clustering methods has rapidly become one of the standard computational approaches in the literature of microarray gene expression data analysis, little attention has been paid to uncertainty in the results obtained.

**Results:**

We present an R/Bioconductor port of a fast novel algorithm for Bayesian agglomerative hierarchical clustering and demonstrate its use in clustering gene expression microarray data. The method performs bottom-up hierarchical clustering, using a Dirichlet Process (infinite mixture) to model uncertainty in the data and Bayesian model selection to decide at each step which clusters to merge.

**Conclusion:**

Biologically plausible results are presented from a well studied data set: expression profiles of *A. thaliana *subjected to a variety of biotic and abiotic stresses. Our method avoids several limitations of traditional methods, for example how many clusters there should be and how to choose a principled distance metric.

## Background

Although the use of clustering methods has rapidly become one of the standard computational approaches in the literature of microarray gene expression data analysis [1-3], little attention has been paid to uncertainty in the results obtained. In clustering, the patterns of expression of different genes across time, treatments, and tissues are grouped into distinct clusters (perhaps organized hierarchically), in which genes in the same cluster are assumed to be potentially functionally related or to be influenced by a common upstream factor. Such cluster structure is often used to aid the elucidation of regulatory networks. Agglomerative hierarchical clustering [1] is one of the most frequently used methods for clustering gene expression profiles. However, commonly used methods for agglomerative hierarchical clustering rely on the setting of some score threshold to distinguish members of a particular cluster from non-members, making the determination of the number of clusters arbitrary and subjective. The algorithm provides no guide to choosing the "correct" number of clusters or the level at which to prune the tree. It is often difficult to know which distance metric to choose, especially for structured data such as gene expression profiles. Moreover, these approaches do not provide a measure of uncertainty about the clustering, making it difficult to compute the predictive quality of the clustering and to make comparisons between clusterings based on different model assumptions (e.g. numbers of clusters, shapes of clusters, etc.). Attempts to address these problems in a classical statistical framework have focused on the use of bootstrapping [4,5] or the use of permutation procedures to calculate local *p*-values for the significance of branching in a dendrogram produced by agglomerative hierarchical clustering [6,7].

A commonly used computational method of *non-hierarchical clustering*, based on measuring Euclidean distance between feature vectors is given by the k-means algorithm [8,9]. However, the k-means algorithm requires the number of clusters to be predefined, and has been shown to be inadequate for describing clusters of unequal size or shape [10], which limits its applicability to many biological datasets.

Bayesian methods provide a principled approach to these types of analyses and are becoming increasingly popular in a variety of problems across many disciplines: clustering stocks with different price dynamics in finance [11], clustering regions with different growth patterns in economics [12], in signal processing applications [13], as well as in computational biology and genetics [14].

Bayesian approaches to hierarchical clustering of gene expression data have been described by Neal [15], who used a Dirichlet diffusion tree model, and by Heard et al. [16,17] who describe a Bayesian model-based approach for clustering time series, based on regression models and nonlinear basis functions. In previous work [18] we have also described an approach to the problem of automatically clustering gene expression profiles, based on the theory of Dirichlet process (i.e. countably infinite) mixtures. However, all this work, like most Bayesian approaches, is based on sampling using Markov Chain Monte Carlo (MCMC) methods. While MCMC has useful theoretical guarantees, its applicability to large post-genomic datasets is limited by its speed.

In this paper, we present an R/Bioconductor port of the fast novel algorithm for Bayesian agglomerative hierarchical clustering (BHC) introduced by Heller and Ghahramani [19]. This algorithm is based on evaluating the marginal likelihoods of a probabilistic model, and may be interpreted as a bottom-up approximate inference method for a Dirichlet process mixture model (DPM). A DPM is a widely used model for clustering [20] which has the interesting property that the prior probability of a new data point joining a cluster is proportional to the number of points already in that cluster. Moreover, with a probability proportional to α/*n *the (*n *+ 1)th data point forms a new cluster. Here α is a hyperparameter controlling the expected number of clusters as a function of the number of data points *n*. The BHC algorithm uses a model based criterion based on the marginal likelihoods of a DPM to merge clusters, rather than using an ad-hoc distance metric. Bayesian hypothesis testing is used to decide which cluster merges increase the tree quality. Importantly, the optimum tree depth is also calculated, resulting in the best number and size of clusters to fit the data.

### Implementation

The BHC algorithm is similar to traditional agglomerative clustering in that it is a one-pass, bottom-up method which initializes each data point in its own cluster and iteratively merges pairs of clusters. However, instead of distance, the algorithm uses a statistical hypothesis test to choose which clusters to merge.

Let  = {***x***^(1)^,..., ***x***^(*n*)^} denote the entire data set, and  the set of data points at the leaves of the subtree *T*_*i*_. The algorithm is initialized with *n *trivial trees, {*T*_*i *_: *i *= 1...*n*} each containing a single data point  = {**x**^(*i*)^}. At each stage the algorithm considers merging all pairs of existing trees. In considering each merge, two hypotheses are compared. The first hypothesis, denoted by  is that all the data in  were in fact generated independently and identically from the *same probabilistic model*, *p*(**x**|*θ*) with unknown parameters *θ*. The alternative hypothesis, denoted by  would be that the data in  has two or more clusters in it.

To evaluate the probability of the data under hypothesis , we need to specify some prior over the parameters of the model, *p*(*θ*|*β*) with hyperparameters *β*. We now have the ingredients to compute the probability of the data  under :

(1)

This calculates the probability that all the data in  were generated from the same parameter values assuming a model of the form *p*(**x**/*θ*). This is a natural model-based criterion for measuring how well the data fit into one cluster.

The probability of the data under the alternative hypothesis,  (if we restrict ourselves to clusterings that partition the data in a manner that is consistent with the subtrees *T*_*i *_and *T*_*j*_, where *T*_*i *_and *T*_*j *_are the two subtrees of *T*_*k*_), is simply a product over the subtrees  where the probability of a data set under a tree (e.g. *p*(|*T*_*i*_)) is defined below. Combining the probability of the data under hypotheses  and , weighted by the prior that all points in  belong to one cluster, , we obtain the marginal probability of the data in tree *T*_*k*_:

(2)

The prior for the merged hypothesis, *π*_*k*_, can be defined such a manner that BHC efficiently computes probabilities of clusterings consistent with the widely used Dirichlet process mixture model. Note that *π*_*k *_is not an estimated parameter but rather a deterministic function of *α *and the number of points in a given subtree. It is computed bottom-up as the tree is built as described in [19].

The posterior probability of the merged hypothesis  is then obtained using Baye's rule:

(3)

If this posterior probability *r*_*k *_> 0.5 it means that the merged hypothesis is more probable than the alternative partitionings and therefore sub-trees should be left intact. Conversely, if *r*_*k *_< 0.5 then the branches constitute separate clusters.

The BHC algorithm is very simple and is shown in the Appendix. Full details of the algorithm and underlying theory, as well as validation results based on synthetic and real non-biological datasets (including comparisons to traditional agglomerative hierarchical clustering using a Euclidean distance metric and average, single and complete linkage methods) can be found in [19].

### Evaluating the Quality of Clustering

For a data set which has labelled classes, it is possible to compare the quality of hierarchical clusterings obtained from different methods to these known classes. However, the literature is notably lacking in quantitative measures of dendrogram quality suitable for use with the BHC algorithm.

For instance, most of the quality indices implemented in the clValid package [21] require a distance metric: since BHC does not use a distance metric these indices are unsuitable for our comparisons. Another commonly used index for measuring the agreement between two clusterings is the adjusted Rand index [22]: large values for the adjusted Rand index mean better agreement between two clusterings. A value of unity would indicate a perfect match between the clustering partition and ground truth, with zero being the expected result for a random partition. However, this index is only really of use if the true clustering structure is known. In most real-world applications of clustering to microarray data, the biological ground truth is unknown. Nevertheless, the adjusted Rand index has been used to evaluate the performance of a variety of clustering algorithms on experimental microarray data by Yeung et al [23]. These authors used a subset of the data described by Ideker et al. [24], a set of 997 mRNA profiles across 20 experiments representing systematic perturbations of the yeast galactose-utilization pathway. A subset of 205 of these genes were assigned to four functional categories (biosynthesis, protein metabolism and modification; energy pathways, carbohydrate metabolism, catabolism; nucleobase, nucleoside, nucleotide and nucleic acid metabolism; transport), based on Gene Ontology (GO) annotations. However, in their supplementary material, Yeung et al. note that since this array data may not fully reflect these functional categories, this classification should be used with caution.

For the purposes of comparison, we have applied our BHC algorithm to this data set, treating the four assigned classes as "ground truth", with the caveat above. The BHC algorithm automatically correctly identifies four classes in the data, as shown by the dendrogram [see Additional file 1]. The adjusted Rand index is 0.955, which is in the upper range of those calculated by Yeung et al. [23]. For comparison, standard hierarchical clustering using average linkage and a correlation distance metric gives an adjusted Rand index of 0.866. The shrinkage correlation coefficient (SCC) of Yao et al. [25], which used the same data set as a benchmark, gives an adjusted Rand index of 0.876.

#### Quality Measures

In order to perform the comparison of two dendrograms produced by different clustering methods, we have devised a new quantitative measure: **DendrogramPurity**, which takes as input a dendrogram tree structure  and a set of class labels  for the leaves of the tree and outputs a single number measuring how "pure" the subtrees of  are with respect to the class labels .

**DendrogramPurity**(, ): where T is a binary tree (dendrogram) with set of leaves ℒ = {1 ..., *L*} and  = {*c*_1_,..., *c*_*L*_} is the set of known class assignments for each leaf. The DendrogramPurity is defined to be the measure obtained from this random process: pick a leaf ℓ uniformly at random. Pick another leaf *j *in the same class, i.e. *c*_ℓ _= *c*_*j*_. Find the smallest subtree containing ℓ and *j*. Measure the fraction of leaves in that subtree which are in the same class, i.e. *c*_ℓ_. The expected value of this fraction is the DendrogramPurity. This measure can be computed efficiently using a bottom up recursion (without needing to resort to sampling). The overall tree purity is 1 if and only if all leaves in each class are contained within some pure subtree.

For each leaf of the tree it also useful to measure how well it fits in with the labels of the leaves in the surrounding subtree. Leaves which do not fit well contribute to decreasing the overall dendrogram purity. These may highlight unusual or misclassified genes, drugs or cell lines. We define the **LeafHarmony **of a leaf ℓ as a measure of how well that leaf fits in.

**LeafHarmony**(ℓ,, ): Pick a random leaf *j *in same class as leaf ℓ, i.e. *c*_*j *_= *c*_ℓ_, *j *≠ ℓ. Find the smallest subtree containing ℓ and *j*. Measure the fraction of leaves in that subtree which are in class *c*_ℓ_. The expected value of this fraction is the LeafHarmony for ℓ and it measures the contribution of that leaf to the DendrogramPurity.

For the case of data sets where there are not clearly defined class labels these measures are not applicable so we have defined a third measure, the **LeafDisparity**, which highlights differences between two hierarchical clusterings (i.e. dendrograms) of the same data. Intuitively, this measures for each leaf of one dedrogram how similar the surrounding subtree is to the corresponding subtree in the other dendrogram. Define the correlation between two sets  and ℛ to be , where |·| denotes the number of elements in a set.  and . Note that a tree  can be converted into a set-of-sets representation  = {*τ*_1_,..., *τ*_*k*_}. For each node *j *in the tree, *τ*_*j *_is the set of the leaves in the subtree descending from *j*. (Thus in a binary tree with *n *leaves contains *n *- 1 non-leaf internal nodes, so *k *= 2*n *- 1).

**LeafDisparity**(ℓ,, ): Convert each tree into a set-of-sets representation. Align the trees: For each set *τ*_*j *_in , find the set *ρ*_*k *_in  such that the correlation is greatest: *r*_*j *_= max_*k*_*c*(*τ*_*j*_, *ρ*_*k*_). For each leaf ℓ find the average of *r*_*j *_over all sets that contain ℓ, calling this (ℓ). If the element ℓ appears in both  and  let its disparity be the minimum of 1 - (ℓ) in either tree. Thus this measure will be symmetric and sensitive to disagreement between the hierarchical clustering given by each tree.

### Software Implementation

The R/Bioconductor port consists of two functions, *bhc *and *WriteOutClusterLabels*. The *bhc *function calls efficient C++ routines for the special case of the BHC algorithm as described in this paper. The algorithm has a computational complexity of order *N*^2 ^for *N *data points, and runs in about 8 minutes on a Macbook Pro 2 GHz laptop for a data-set of size 880 and dimensionality 31 (i.e. the NASC data set used in this paper). The reverse clustering (i.e. size 31 with dimensionality 880) runs in approximately a minute. For runtimes for data sets of various sizes [see Additional file 2].

The *WriteOutClusterLabels *function outputs the resulting cluster labels to an ASCII file. Because the *bhc *function outputs its results in a standard R *dendrogram *object, a graphical representation of the output can be obtained by calling the standard R *plot *function. A 2D heat-map visualization of the clustering can be generated using the standard R function *heatmap*.

In our model the hyperparameters are the concentration parameter, *α*, which controls the distribution of the prior weight assigned to each cluster in the DPM, and is directly related to the expected number of clusters, and the hyperparameters, *β*, of the probabilistic model defining each component of the mixture. The concentration parameter, *α*, was fixed to a small, positive value (0.001). The hyperparameters for the individual mixture component (Dirichlet) priors *β *are scaled by a single additional hyperparameter, giving the data model greater flexibility. This additional hyperparameter was determined by optimising the overall model Evidence (marginal likelihood), using a combination of golden section search and successive parabolic interpolation (as implemented in the R function *optimize*). The unscaled *β *hyperparameters were set by using the whole data-set as a measure of the relative proportions of each discrete value for each gene.

### Application to Microarray Data

We illustrate our methods with application to a published data set of GeneChip expression profiles of *A. thaliana *subjected to a variety of biotic and abiotic stresses, derived from the AtGenExpress consortium (NASC), identical to that used by Torres-Zabala et al. [26]. The expression profiles were selected, normalized and interpreted by the GC content-adjusted robust multi-array algorithm (GCRMA) [27] exactly as in the original manuscript. Continuous transcript levels were discretised into three levels (unchanged, under- or over-expressed) by dividing the levels at fixed quantiles for each given gene. This makes our analyses more robust to any experimental systematics, as well as simplifying the algorithm by using multinomial distributions and their conjugate Dirichlet priors. By discretizing mRNA levels we model the important qualitative changes in mRNA levels without making strong unjustifiable assumptions (e.g. Gaussianity) about the form of the noise in microarray experiments. We note that such an approach has also been used by other workers in the field [28]. In order to identify the optimal discretization thresholds we utilized the following procedure. The discretization threshold is parameterised via the quantiles, *x*, of the data for a given gene, such that the data counts are distributed in the proportions *x *: (1 - 2*x*): *x*. We can then optimise *x *jointly over all the genes by running the BHC algorithm for different *x *values (and hence discretisations) and using the lower bound on the overall model Evidence, modified to account for the above parameterisation by dividing the Evidence by the relevant bin width for each data point. Evidence values and the optimal value for the hyperparameter mentioned in the previous section are shown [see Additional files 3 and 4]. These results indicate that the optimal quantitles for the discretization of this data set are 20/60/20 and 25/50/25 for the experiment and gene clustering, respectively.

## Results

**Figure 1:**
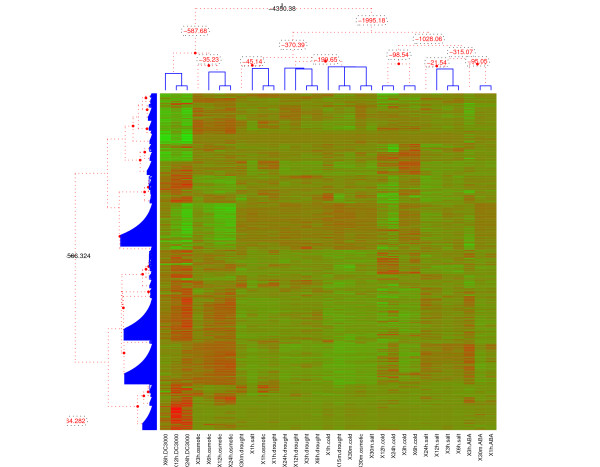
**Clustering of 880 genes and 31 conditions of A. thaliana**. Clustering of 880 genes and 31 conditions of *A. thaliana*, subjected to a variety of biotic and abiotic stresses (as used by [26]). Shown are transcript profile clustering (left), condition clustering (above and below) and the corresponding 2D heat map, aligned with the 1D dendrograms. Red dashed lines are merges our algorithm prefers not to make. The numbers on the branches are the log odds for merging ().

Clustering of the Arabidopsis genes and experimental conditions was carried out using our BHC algorithm and a biologically plausible clustering pattern was observed (Figure 1). This was compared to the conventional agglomerative hierarchical clustering of the same data carried out by de Torres-Zabala et al. [26], using an uncentred correlation coefficient as a distance metric and complete linkage. We observed that the essential features of the hierarchical clustering of experimental conditions were reproduced, but with more specific clusters as evidenced by the DendrogramPurity measure of 0.968 (BHC) versus 0.473 (agglomerative hierarchical clustering). LeafHarmony measures for the BHC clustering [see Additional file 5] show that most leaves have a value of 1.0, indicating the consistency of the clusters produced. In particular, we observed specific clusters for drought, osmotic stress and salt. In the case of pathogen infection (DC3000) and the phytohormone abscisic acid (ABA) treatment, we find that each group of experiments forms a well-defined cluster. We note that in the clustering of de Torres-Zabala et al. [26] only two of the ABA experiments (30 min and 1 h) cluster at the lowest level, and splitting the dendrogram at this level places the ABA 3 h experiment in a separate cluster with salt and osmotic stress experiments. The clustering produced by BHC thus seems more intuitive, with the ABA 3 h experiment appearing unconnected in the dendrogram. An advantage of the BHC method over conventional hierarchical clustering is that Bayesian hypothesis testing is used to decide which clusters to merge.

The overall dendrogram structures, are, however, demonstrably different, as evidenced by the comparatively low values of LeafDisparity [see Additional file 6] and the adjusted Rand indices of 0.299 for the gene clusters and 0.325 for the experiment clusters.

For the genes, we find that BHC produces a clustering of finer granularity; for instance, genes highlighted in clusters I-IV in de Torres-Zabala et al. [26] are split between a number of smaller clusters (see Additional files 7 and 8). Most of the genes in clusters I and II are divided between our clusters 5 and 7. Cluster 5 contains 22 out of the 28 genes in cluster II, including six PP2Cs, NCED3 and three NAC domain transcription factors, all of which are known to be regulated by ABA. Genes in cluster III are all in BHC cluster 16, which is enriched with Gene Ontology annotations indicating chloroplast function (see below). To further validate the quality of the clusters produced by BHC we have analyzed the statistically significantly over-represented GO annotations related to a given cluster of genes. The probability that this over-representation is not found by chance can be calculated by the use of a hypergeometric test, implemented in the R/Bioconductor package *GOstats *[29]. Because of the effects of multiple testing, a subsequent correction of the *p*-values is necessary. We apply a Bonferroni correction, which gives a conservative (and easily calculated) correction for multiple testing. We extract the lowest levels of the ontology graphs using the *GOstats *command 'sigCategories'. In the supplementary material we show the lowest level GO annotations for the BHC clusters which are significant at a Bonferroni-corrected *p*-value of 0.05. We compared the enriched GO annotations for the BHC clusters to those from the agglomerative hierarchical clustering of Torres-Zabala et al. (see Additional files 9 and 10). To quantify this comparison, we calculated the Biological Homogeneity Index (BHI) of Datta and Datta [30] as implemented in the clValid package of Brock et al. [21]. This index provides a measure the 'biological meaning' of clusters based on the homogeneity of functional classes represented by the GO annotations. Taking the number of clusters to be 29, as found by BHC, we calculate a BHI of 0.179 (BHC) versus 0.161 (agglomerative hierarchical clustering), indicating more biologically homogeneous clusters in the former case.

As mentioned above, we observe some overlap between significant GO annotations for two of these clusters (II with BHC cluster 5; III with BHC cluster 16). However, many biologically significant terms are enriched only in the BHC clusters (for example camalexin biosynthesis in BHC cluster 29), indicating that the BHC clusters represent a more refined view of the data, which enables processes important in defence to be identified. This can be illustrated by examining the GO groupings of the BHC clusters that are intuitively meaningful in the context of plant-microbe interactions.

For example, cluster 16 comprises a major cluster of genes associated with chloroplast function and chlorophyll biosynthesis. Chloroplasts are emerging as a key target of bacterial effector function [31].

Interestingly, cluster 10 is strongly biased towards genes involved in ion homeostasis, and changes in ion fluxes represent the earliest physiological changes associated with plant defences. Rapid ion changes are often associated with changes in phosphorylation status of transporters, and cluster 5 is over-represented by cellular components associated with phosphorylation. Reconfiguration of secondary metabolism is central to the ability to modify plant defences. Notably, clusters 29 and 6 elegantly capture pathway components of indolic and jasmonic acid metabolism. Within this context, cluster 19 is worthy of further investigation. Members of cluster 19 directly impact upon the secondary metabolism defined in clusters 29 and 6 above. Thus the BHC approach may have revealed a set of co-regulated genes whose biological activity is responsible for activating the biosynthetic networks highlighted in clusters 29 and 6.

Experiments to address this hypothesis are currently underway.

## Conclusion

We have presented an R/Bioconductor port of a fast novel algorithm for Bayesian agglomerative hierarchical clustering and have demonstrated its use in clustering gene expression microarray data. Biologically plausible results are presented from a well studied data set: expression profiles of *A. thaliana *subjected to a variety of biotic and abiotic stresses. The BHC approach has identified a new avenue of research not revealed by the previous clustering analyses of this data. The use of a probabilistic approach to model uncertainty in the data, and Bayesian model selection to decide at each step which clusters to merge, avoids several limitations of traditional clustering methods, such as how many clusters there should be and how to choose a principled distance metric. Extensions of the algorithm described here are straightforward for other distributions in the exponential family, such as Gaussians [19], which may be useful when such distributions are well justified for the data in question.

## Availability and requirements

Available under the Gnu GPL from  and through the Bioconductor website. Online supplementary data is available at the journal's web site.

## Authors' contributions

RS and YX wrote the code. RS carried out the computational analyses. KH and ZG developed the algorithm. WMT and MG provided the Arabidopsis data. KD, MG, ZG and DLW wrote the paper. ZG and DLW directed the research. All authors read and approved the final version of the manuscript.

## Appendix

   **input: **data  = {**x**^(1) ^... **x**^(*n*)^}, model *p*(**x**|θ), prior *p*(*θ*|*β*)

   **initialize: **number of clusters *c *= *n*, and  = {**x**^(*i*)^} for *i *= 1 ...*n*

   **while ***c *> 1 **do**

      Find the pair  and  with the highest probability of the merged hypothesis:



      Merge , *T*_*k *_← (*T*_*i*_, *T*_*j*_)

      Delete *D*_*i *_and *D*_*j*_, *c *← *c *- 1

   **end while**

   **output: **Bayesian mixture model where each tree node is a mixture component

The tree can be cut at points where *r*_*k *_< 0.5

Algorithm 1: Bayesian Hierarchical Clustering Algorithm

## Supplementary Material

Additional file 1 **Figure 2**. Gene clustering dendrogram of a subset of the Ideker et al. data, showing leaf harmony values Click here for file

Additional file 2 **Table 1 – Speed-trial of the BHC algorithm**. Trials were based on the NASC data (880 genes, 31 features), clustering over genes. In each case, the data were duplicated or a subset of genes taken as appropriate to get the required number genes and features. All trials were run on a single 2 GHz CPU core on a Macbook Pro laptop. Click here for file

Additional file 3 **Table 2**. Data discretisation for NASC experiment clustering Click here for file

Additional file 4 **Table 3**. Data discretisation for NASC gene clustering Click here for file

Additional file 5 **Figure 3**. Condition clustering dendrogram for the NASC data. Click here for file

Additional file 6 **LeafDisparity values for the NASC experiments**. The BHC clustering dendrogram is compared to a standard hierarchical method using uncentred correlation coefficients and complete linnkage. Click here for file

Additional file 7 **Figure 4**. Gene clustering dendrogram for the NASC data. Click here for file

Additional file 8 **BHC cluster membership**. BHC cluster membership Click here for file

Additional file 9 **GO annotations for BHC clusters**. Statistically significantly over-represented GO annotations for BHC clusters (Bonferroni-corrected *p*-value < 0.05) Click here for file

Additional file 10 **GO annotations for agglomerative hierarchical clustering**. Statistically significantly over-represented GO annotations for clusters manually identified from agglomerative hierarchical clustering (Bonferroni-corrected *p*-value < 0.05) Click here for file
