# Supplementary figures and images for: R/BHC: fast Bayesian hierarchical clustering for microarray data
Source: BMC Bioinformatics. 2009 Aug 6;10:242. doi: 10.1186/1471-2105-10-242 (PMC2736174; doi:10.1186/1471-2105-10-242)

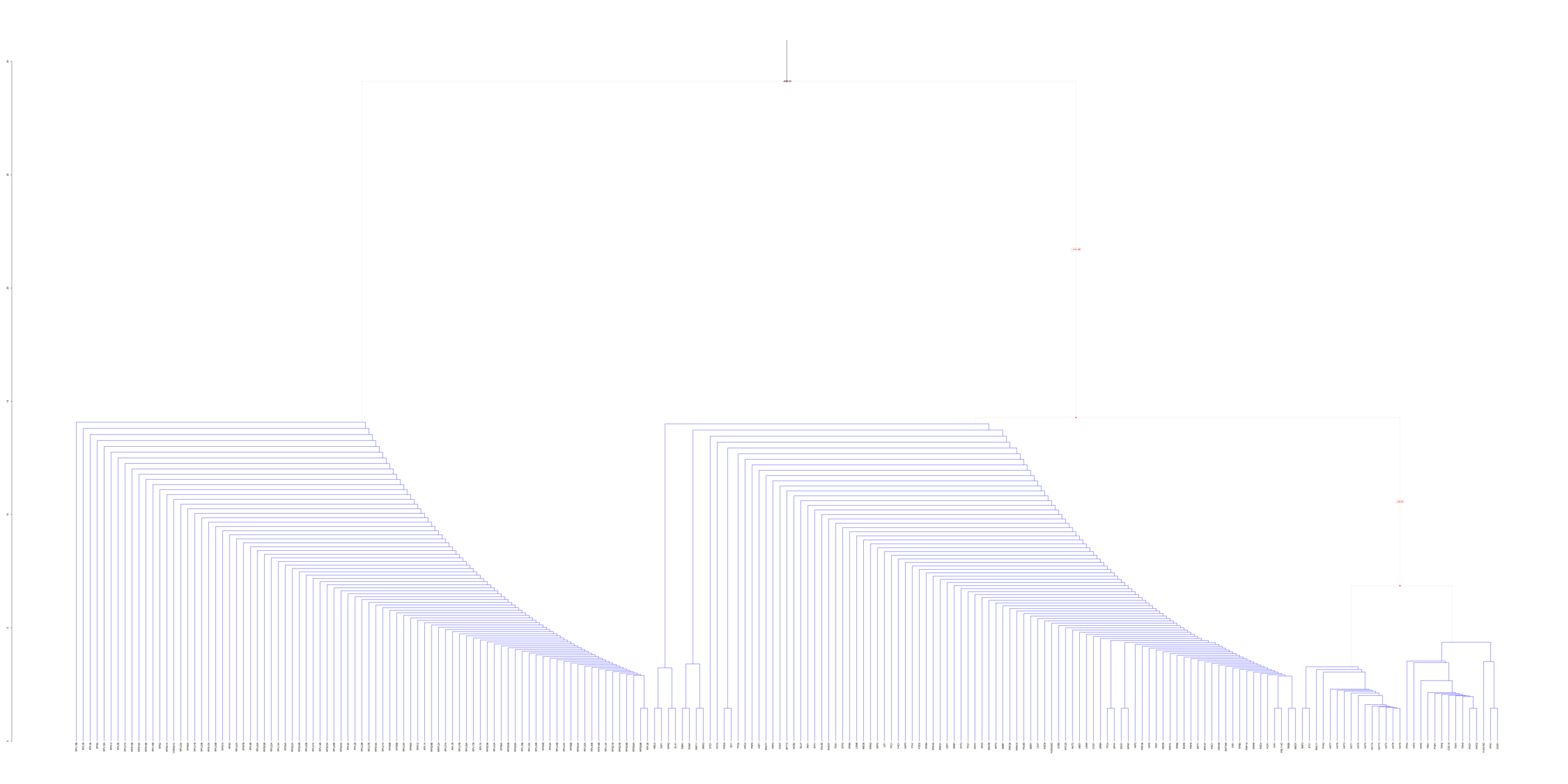

Supplement: Additional file 1 — Figure 2. Gene clustering dendrogram of a subset of the Ideker et al. data, showing leaf harmony values [file 1471-2105-10-242-S1.pdf]

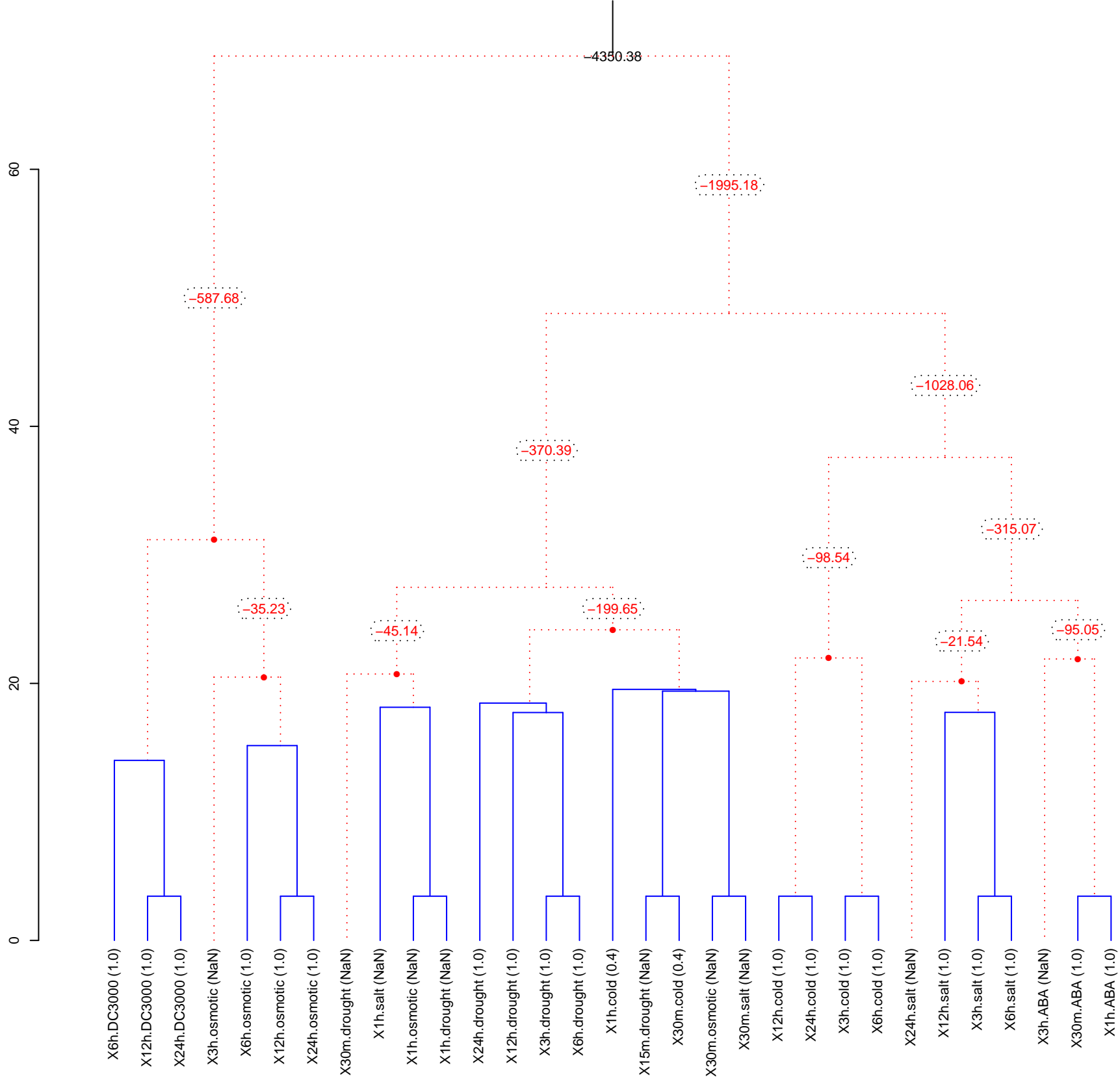

Supplement: Additional file 5 — Figure 3. Condition clustering dendrogram for the NASC data. [file 1471-2105-10-242-S5.pdf]
