# Supplementary material for: R/BHC: fast Bayesian hierarchical clustering for microarray data
Source: BMC Bioinformatics. 2009 Aug 6;10:242. doi: 10.1186/1471-2105-10-242 (PMC2736174; doi:10.1186/1471-2105-10-242)
Supplement: Additional file 2 — Table 1 – Speed-trial of the BHC algorithm. Trials were based on the NASC data (880 genes, 31 features), clustering over genes. In each case, the data were duplicated or a subset of genes taken as appropriate to get the required number genes and features. All trials were run on a single 2 GHz CPU core on a Macbook Pro laptop. [file 1471-2105-10-242-S2.pdf]

	440 genes	880 genes	1760 genes
31 features	2 mins	8 mins	34 mins
62 features	4 mins	16 mins	68 mins
124 features	7 mins	20 mins	146 mins

Table 1: Speed-trial of the BHC algorithm. Trials were based on the NASC data (880 genes, 31 features), clustering over genes. In each case, the data were duplicated or a subset of genes taken as appropriate to get the required number genes, features. All trails were run on a single 2 GHz CPU core on a Macbook Pro laptop.
