# Supplementary material for: R/BHC: fast Bayesian hierarchical clustering for microarray data
Source: BMC Bioinformatics. 2009 Aug 6;10:242. doi: 10.1186/1471-2105-10-242 (PMC2736174; doi:10.1186/1471-2105-10-242)
Supplement: Additional file 3 — Table 2. Data discretisation for NASC experiment clustering [file 1471-2105-10-242-S3.pdf]

Quantiles	log-Evidence	hyperparameter
10/80/10	-3.00×10^4	2.33
15/70/15	-2.83×10^4	2.18
19/62/19	-2.72×10^4	2.40
20/60/20	-2.68×10^4	1.84
21/58/21	-2.68×10^4	2.56
22/56/22	-2.68×10^4	2.56
23/54/23	-2.69×10^4	2.56
25/50/25	-2.69×10^4	2.69

Table 1: Data discretisation for NASC experiment clustering.
