# Supplementary material for: R/BHC: fast Bayesian hierarchical clustering for microarray data
Source: BMC Bioinformatics. 2009 Aug 6;10:242. doi: 10.1186/1471-2105-10-242 (PMC2736174; doi:10.1186/1471-2105-10-242)
Supplement: Additional file 4 — Table 3. Data discretisation for NASC gene clustering [file 1471-2105-10-242-S4.pdf]

Quantiles	log-Evidence	hyperparameter
10/80/10	-2.76×10^4	3.17
15/70/15	-2.54×10^4	2.33
20/60/20	-2.37×10^4	2.33
23/54/23	-2.36×10^4	2.33
25/50/25	-2.36×10^4	2.33
27/46/27	-2.40×10^4	1.84
30/40/30	-2.44×10^4	1.84

Table 1: Data discretisation for NASC gene clustering.
