# Supplementary material for: R/BHC: fast Bayesian hierarchical clustering for microarray data
Source: BMC Bioinformatics. 2009 Aug 6;10:242. doi: 10.1186/1471-2105-10-242 (PMC2736174; doi:10.1186/1471-2105-10-242)
Supplement: Additional file 6 — LeafDisparity values for the NASC experiments. The BHC clustering dendrogram is compared to a standard hierarchical method using uncentred correlation coefficients and complete linnkage. [file 1471-2105-10-242-S6.pdf]

Experiment	disparity
6h.DC3000	0.1071429
12h.DC3000	0.08571429
24h.DC3000	0.08571429
30m.ABA	0.1952276
1h.ABA	0.1952276
3h.ABA	0.2277655
30m.osmotic	0.1661290
1h.osmotic	0.2078053
3h.osmotic	0.1071429
6h.osmotic	0.1357143
12h.osmotic	0.1130952
24h.osmotic	0.1130952
15m.drought	0
30m.drought	0.2112903
1h.drought	0.2402714
3h.drought	0.1786600
6h.drought	0.1786600
12h.drought	0.2329032
24h.drought	0.2329032
30m.salt	0.2320789
1h.salt	0.2320789
3h.salt	0.1708242
6h.salt	0.1708242
12h.salt	0.1476086
24h.salt	0.1722100
30m.cold	0
1h.cold	0.1784946
3h.cold	0.1765553
6h.cold	0.1765553
12h.cold	0.1303489
24h.cold	0.1303489

Table 1: Disparity values for the NASC experiments. The BHC clustering dendrogram is compared to a standard hierarchical method using uncentred correlation coefficients and complete linnkage.
