# Supplementary material for: R/BHC: fast Bayesian hierarchical clustering for microarray data
Source: BMC Bioinformatics. 2009 Aug 6;10:242. doi: 10.1186/1471-2105-10-242 (PMC2736174; doi:10.1186/1471-2105-10-242)

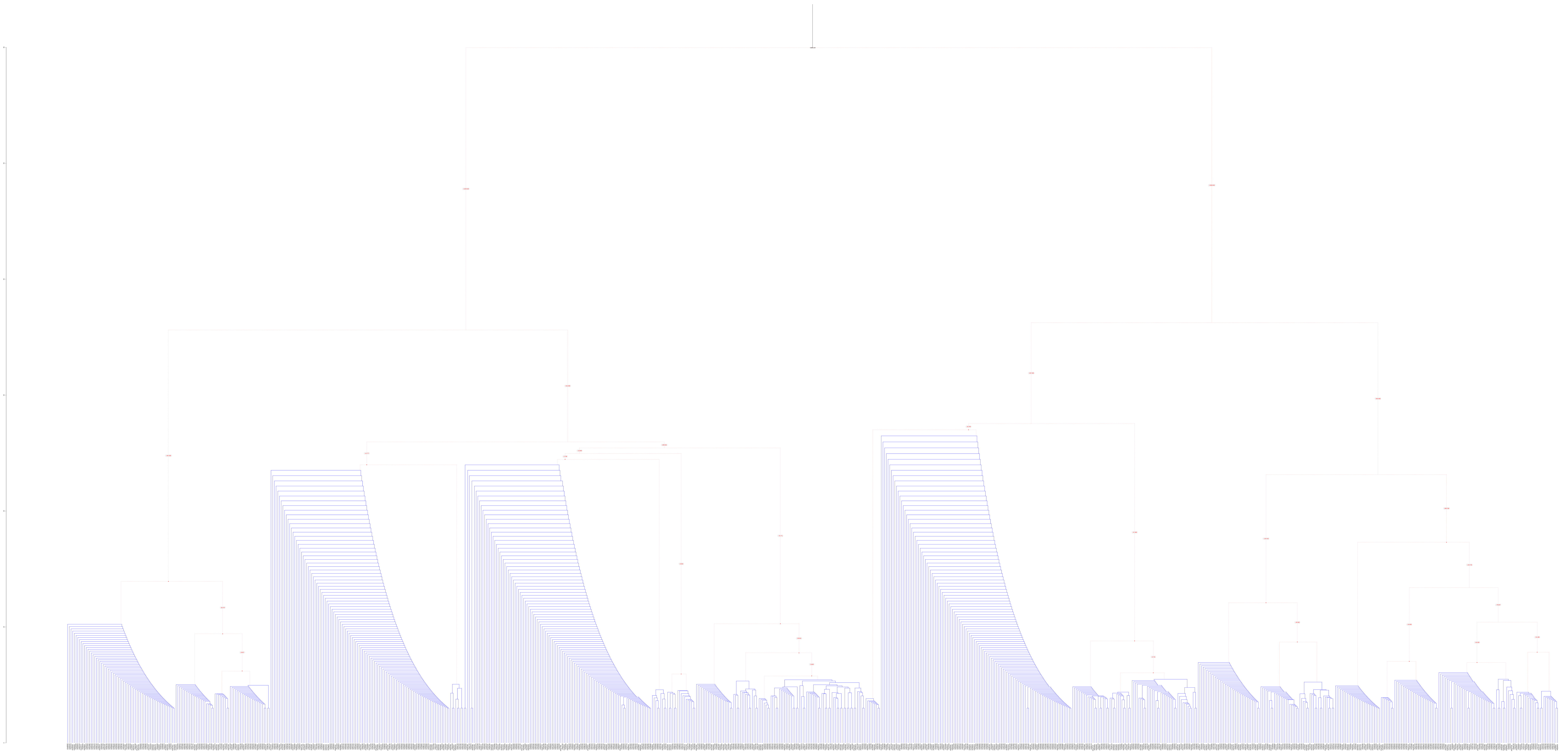

Supplement: Additional file 7 — Figure 4. Gene clustering dendrogram for the NASC data. [file 1471-2105-10-242-S7.pdf]
