# Supplementary material for: R/BHC: fast Bayesian hierarchical clustering for microarray data
Source: BMC Bioinformatics. 2009 Aug 6;10:242. doi: 10.1186/1471-2105-10-242 (PMC2736174; doi:10.1186/1471-2105-10-242)
Supplement: Additional file 9 — GO annotations for BHC clusters. Statistically significantly over-represented GO annotations for BHC clusters (Bonferroni-corrected p-value < 0.05) [file 1471-2105-10-242-S9.pdf]

Cluster	GO term (p-value)
1	
2	C-22 sterol desaturase activity (1.40×10^{-2})
	detection of fungus (2.34×10^{-2})
3	cytidine deaminase activity (3.51×10^{-3})
	response to singlet oxygen (4.58×10^{-3})
	nucleoside catabolic process (6.11×10^{-3})
	cytidine deamination (6.11×10^{-3})
	defense response signaling pathway, resistance gene-independent (6.11×10^{-3})
	cytidine metabolic process (6.11×10^{-3})
	pyrimidine ribonucleoside catabolic process (6.11×10^{-3})
	pyrimidine nucleoside metabolic process (7.63×10^{-3})
	defense response signaling pathway, resistance gene-dependent (8.4×10^{-3})
	response to stress (1.10×10^{-2})
	ribonucleoside metabolic process (1.22×10^{-2})
	intrinsic to endoplasmic reticulum membrane (1.22×10^{-2})
	magnesium ion binding (1.25×10^{-2})
	activation of immune response (1.68×10^{-2})
	positive regulation of immune system process (1.68×10^{-2})
	positive regulation of innate immune response (1.68×10^{-2})
	systemic acquired resistance (2.06×10^{-2})
	regulation of immune response (2.06×10^{-2})
	plant-type hypersensitive response (2.36×10^{-2})
	positive regulation of response to stimulus (3.20×10^{-2})
	monooxygenase activity (3.26×10^{-2})
	hydrolase activity, acting on carbon-nitrogen (but not peptide) bonds (3.37×10^{-2})
	regulation of defense response (3.5×10^{-2})
	microsome (3.67×10^{-2})
	membrane fraction (4.54×10^{-2})
4	
5	protein serine/threonine phosphatase complex (9.9×10^{-6})
	protein serine/threonine phosphatase activity (1.79×10^{-5})
	protein amino acid dephosphorylation (6.12×10^{-4})
	phosphatase activity (8.16×10^{-4})
	guanosine tetraphosphate metabolic process (3.56×10^{-3})
	hydrolase activity, acting on ester bonds (5.29×10^{-3})
	purine ribonucleoside metabolic process (1.24×10^{-2})
	negative regulation of abscisic acid mediated signaling (4.54×10^{-2})
6	response to insect (2.04×10^{-3})
	jasmonic acid and ethylene-dependent systemic resistance (6.71×10^{-3})
	response to starvation (8.33×10^{-3})
	response to extracellular stimulus (1.16×10^{-2})
	response to ethylene stimulus (2.64×10^{-2})
	response to jasmonic acid stimulus (2.64×10^{-2})
	response to external stimulus (2.69×10^{-2})

	innate immune response (3.04×10^{-2})
	immune system process (3.28×10^{-2})
	calcium ion binding (3.98×10^{-2})
7	
8	autophagy (3.2×10^{-2})
9	lactoylglutathione lyase activity (1.02×10^{-3})
	response to sucrose stimulus (5.19×10^{-3})
	aging (1.05×10^{-2})
	response to carbohydrate stimulus (1.08×10^{-2})
	endomembrane system (1.58×10^{-2})
	lyase activity (4.53×10^{-2})
	hydrolase activity, hydrolyzing O-glycosyl compounds (4.98×10^{-2})
10	cellular response to sucrose starvation (2.44×10^{-3})
	asparagine synthase (glutamine-hydrolyzing) activity (3.07×10^{-3})
	water homeostasis (9.77×10^{-3})
	acyl carrier activity (1.13×10^{-2})
	cellular potassium ion homeostasis (1.22×10^{-2})
	response to absence of light (2.68×10^{-2})
	monovalent inorganic cation homeostasis (2.68×10^{-2})
	sodium:hydrogen antiporter activity (3.26×10^{-2})
	solute:hydrogen antiporter activity (4.99×10^{-2})
11	immune system process (6.89×10^{-5})
	drug transporter activity (7.76×10^{-4})
	negative regulation of defense response (8.52×10^{-4})
	plasma membrane (1.81×10^{-2})
	auxin efflux (1.96×10^{-2})
	xenobiotic-transporting ATPase activity (2.54×10^{-2})
	multidrug efflux pump activity (2.54×10^{-2})
	ATPase activity (3.2×10^{-2})
	response to humidity (3.91×10^{-2})
	positive regulation of cellular defense response (3.91×10^{-2})
	positive regulation of cell size (3.91×10^{-2})
12	sphingolipid delta-4 desaturase activity (2.68×10^{-3})
	methionine adenosyltransferase activity (1.07×10^{-2})
	positive regulation of anthocyanin metabolic process (1.59×10^{-2})
	S-adenosylmethionine biosynthetic process (3.18×10^{-2})
	double-stranded DNA binding (3.48×10^{-2})
	positive regulation of flavonoid biosynthetic process (3.97×10^{-2})
	lipid transporter activity (4.28×10^{-2})
13	NAD+ ADP-ribosyltransferase activity (1.17×10^{-3})
	oxygen and reactive oxygen species metabolic process (7.52×10^{-3})
	response to salt stress (4.01×10^{-2})
14	
15	endoplasmic reticulum (4.99×10^{-8})
	protein folding (3.96×10^{-5})
	ATP binding (1.49×10^{-3})

	adenyl nucleotide binding (1.80×10^{-3})
	purine ribonucleotide binding (3.19×10^{-3})
	nucleotide binding (7.03×10^{-3})
	regulation of meristem growth (1.57×10^{-2})
	protein secretion (1.71×10^{-2})
	regulation of meristem organization (1.99×10^{-2})
	endoplasmic reticulum lumen (2.07×10^{-2})
	regulation of growth (3.56×10^{-2})
	cytoplasm (4.26×10^{-2})
16	chloroplast thylakoid membrane (2.13×10^{-5})
	thylakoid membrane (2.74×10^{-5})
	plastid thylakoid (8.4×10^{-5})
	photosynthetic membrane (1.79×10^{-4})
	chloroplast (2.94×10^{-4})
	intracellular organelle part (2.81×10^{-2})
	cytoplasmic part (2.99×10^{-2})
17	autophagic vacuole (1.32×10^{-2})
	outward rectifier potassium channel activity (3.82×10^{-2})
	phosphatidylinositol-4,5-bisphosphate 5-phosphatase activity (4.36×10^{-2})
	autophagy (4.74×10^{-2})
18	nitrate reductase activity (7.8×10^{-4})
	prephenate dehydratase activity (2.34×10^{-3})
	arogenate dehydratase activity (2.34×10^{-3})
	nitric oxide biosynthetic process (4.28×10^{-3})
	L-phenylalanine biosynthetic process (8.55×10^{-3})
	nitrate assimilation (9.98×10^{-3})
	nitrogen compound metabolic process (2.00×10^{-2})
	defense response to fungus, incompatible interaction (2.14×10^{-2})
	defense response to bacterium, incompatible interaction (3.42×10^{-2})
	carbon-oxygen lyase activity (4.3×10^{-2})
19	jasmonate O-methyltransferase activity (1.27×10^{-2})
	6-phosphogluconolactonase activity (2.54×10^{-2})
	beta-carotene hydroxylase activity (2.54×10^{-2})
	1,2-diacylglycerol 3-beta-galactosyltransferase activity (3.8×10^{-2})
20	protein serine/threonine phosphatase activity (4.73×10^{-3})
21	extracellular matrix structural constituent (1.09×10^{-2})
	ubiquitin ligase complex (2.54×10^{-2})
	protein ubiquitination (3.24×10^{-2})
	extracellular matrix organization and biogenesis (3.28×10^{-2})
	peptide receptor activity (4.37×10^{-2})
22	flavonol biosynthetic process (7.68×10^{-5})
	caffeate O-methyltransferase activity (4.78×10^{-3})
	flavonoid metabolic process (1.24×10^{-2})
	pigment metabolic process (3.32×10^{-2})
	beta-amylase activity (4.29×10^{-2})
23	substrate-specific transmembrane transporter activity (1.25×10^{-3})

	cation transmembrane transporter activity (1.76×10^{-3})
	anion transport (6.96×10^{-3})
	transporter activity (1.71×10^{-2})
	electron carrier activity (2.47×10^{-2})
	localization (2.58×10^{-2})
	rhamnogalacturonan II biosynthetic process (3.66×10^{-2})
	UDP-xylosyltransferase activity (4.29×10^{-2})
	metal ion transmembrane transporter activity (4.61×10^{-2})
24	endomembrane system (1.57×10^{-2})
	protein ubiquitination (2.39×10^{-2})
	defense response to bacterium (2.48×10^{-2})
25	endomembrane system (1.62×10^{-4})
	electron carrier activity (2.58×10^{-3})
	kinase activity (5.23×10^{-3})
	defense response to bacterium, incompatible interaction (1.26×10^{-2})
	immune system process (2.57×10^{-2})
	positive regulation of biosynthetic process (4.46×10^{-2})
26	kinase activity (2.21×10^{-4})
	protein amino acid phosphorylation (3.04×10^{-4})
	oligopeptide transport (5.59×10^{-4})
	phosphate metabolic process (7.26×10^{-4})
	oligopeptide transporter activity (1.48×10^{-3})
	endomembrane system (4.22×10^{-3})
	protein modification process (8.92×10^{-3})
	transferase activity (2.23×10^{-2})
27	hydrogen peroxide-mediated programmed cell death (3.06×10^{-3})
	ADP-ribose diphosphatase activity (3.51×10^{-3})
	NAD binding (1.05×10^{-2})
28	urea transmembrane transporter activity (1.17×10^{-3})
	cyclic-nucleotide phosphodiesterase activity (2.34×10^{-3})
	isochorismate synthase activity (2.34×10^{-3})
	protein disulfide isomerase activity (4.68×10^{-3})
	salicylic acid biosynthetic process (8.55×10^{-3})
	solute:sodium symporter activity (1.05×10^{-2})
	phylloquinone biosynthetic process (1.43×10^{-2})
	tRNA splicing (1.71×10^{-2})
	cellular response to nitrogen starvation (1.71×10^{-2})
	vitamin K metabolic process (1.71×10^{-2})
	transporter activity (2.52×10^{-2})
	phosphate transport (3.42×10^{-2})
	intramolecular oxidoreductase activity (3.50×10^{-2})
	negative regulation of defense response (3.7×10^{-2})
	fat-soluble vitamin biosynthetic process (3.7×10^{-2})
	intramolecular transferase activity (4.07×10^{-2})
	quinone cofactor biosynthetic process (4.27×10^{-2})
29	glycerol kinase activity (1.95×10^{-3})

tryptophan catabolic process (1.14×10^{-2})
biogenic amine catabolic process (1.14×10^{-2})
indole derivative catabolic process (1.14×10^{-2})
catabolic process (1.73×10^{-2})
chitinase activity (2.73×10^{-2})
camalexin biosynthetic process (2.85×10^{-2})
response to microbial phytotoxin (3.42×10^{-2})
indole phytoalexin metabolic process (3.42×10^{-2})
phytoalexin biosynthetic process (3.42×10^{-2})
glycerol catabolic process (3.99×10^{-2})
response to molecule of bacterial origin (4.56×10^{-2})
indoleacetic acid biosynthetic process (4.56×10^{-2})
