# Supplementary material for: R/BHC: fast Bayesian hierarchical clustering for microarray data
Source: BMC Bioinformatics. 2009 Aug 6;10:242. doi: 10.1186/1471-2105-10-242 (PMC2736174; doi:10.1186/1471-2105-10-242)
Supplement: Additional file 10 — GO annotations for agglomerative hierarchical clustering. Statistically significantly over-represented GO annotations for clusters manually identified from agglomerative hierarchical clustering (Bonferroni-corrected p-value < 0.05) [file 1471-2105-10-242-S10.pdf]

Cluster	GO term (p-value)
1	response to wounding (6.2×10^{-4})
	cellular amino acid and derivative metabolic process (1.12×10^{-3})
	lignin biosynthetic process (1.72×10^{-3})
	trans-cinnamate 4-monooxygenase activity (2.22×10^{-3})
	quercetin 3-O-methyltransferase activity (2.22×10^{-3})
	myricetin 3'-O-methyltransferase activity (2.22×10^{-3})
	ferulate 5-hydroxylase activity (2.22×10^{-3})
	caffeate O-methyltransferase activity (2.22×10^{-3})
	response to stimulus (5.05×10^{-3})
	response to radiation (1.62×10^{-2})
	beta-fructofuranosidase activity (2.43×10^{-2})
	cell wall (2.57×10^{-2})
	4-coumarate-CoA ligase activity (2.65×10^{-2})
	catalytic activity (3.92×10^{-2})
	flavonol biosynthetic process (4.89×10^{-2})
2	acyl-CoA thioesterase activity (4.34×10^{-2})
3	protein disulfide isomerase activity (1.43×10^{-4})
	intramolecular oxidoreductase activity (1.28×10^{-3})
	cell wall (1.69×10^{-3})
	cyclic-nucleotide phosphodiesterase activity (6.04×10^{-3})
	lytic vacuole within protein storage vacuole (7.45×10^{-3})
	storage vacuole (2.98×10^{-2})
4	electron carrier activity (3.99×10^{-4})
	chitinase activity (6.02×10^{-3})
	intracellular ligand-gated ion channel activity (1.51×10^{-2})
	multi-organism process (2.21×10^{-2})
	apoplast (2.60×10^{-2})
	response to bacterium (2.76×10^{-2})
	ligand-gated channel activity (3.22×10^{-2})
	endomembrane system (3.27×10^{-2})
	cell wall (4.45×10^{-2})
5	extracellular matrix structural constituent (1.05×10^{-2})
	shikimate O-hydroxycinnamoyltransferase activity (1.05×10^{-2})
	quinate O-hydroxycinnamoyltransferase activity (1.05×10^{-2})
	hydroxycinnamoyltransferase activity (3.15×10^{-2})
6	phosphate transport (1.46×10^{-3})
	carbohydrate transmembrane transporter activity (2.26×10^{-3})
	solute:cation symporter activity (2.32×10^{-3})
	anion transport (3.45×10^{-2})
	sugar:hydrogen symporter activity (3.77×10^{-2})
7	response to stimulus (7.93×10^{-3})
	endoplasmic reticulum lumen (1.28×10^{-2})
	nitrate transmembrane transporter activity (1.57×10^{-2})
	protein amino acid phosphorylation (2.3×10^{-2})

8	chloroplast (6.11×10^{-6})
	chloroplast thylakoid membrane (1.83×10^{-4})
	thylakoid membrane (2.33×10^{-4})
	anchored to plasma membrane (2.62×10^{-4})
	plant-type cell wall (4.63×10^{-4})
	plastid thylakoid (5.38×10^{-4})
	chloroplast stroma (1.39×10^{-3})
	cytoplasm (5.16×10^{-3})
	external encapsulating structure (1.72×10^{-2})
	organelle part (2.34×10^{-2})
	unfolded protein binding (3×10^{-2})
	intracellular membrane-bounded organelle (4.3×10^{-2})
9	oligopeptide transporter activity (3.17×10^{-3})
	cellular metal ion homeostasis (5.91×10^{-3})
	pectinesterase inhibitor activity (6.63×10^{-3})
	oligopeptide transport (9.39×10^{-3})
	cellular ion homeostasis (10×10^{-3})
	polygalacturonase activity (1.01×10^{-2})
	cation homeostasis (1.01×10^{-2})
	chemical homeostasis (1.26×10^{-2})
	cellular homeostasis (1.80×10^{-2})
	enzyme regulator activity (3.57×10^{-2})
10	chloroplast (5.03×10^{-3})
	chloroplast thylakoid membrane (3.61×10^{-2})
	thylakoid membrane (4.02×10^{-2})
	low affinity phosphate transmembrane transporter activity (4.68×10^{-2})
11	photosynthetic electron transport in photosystem II ($NaN \times 10^{-Inf}$)
	protein import into chloroplast thylakoid membrane ($NaN \times 10^{-Inf}$)
	thylakoid membrane organization ($NaN \times 10^{-Inf}$)
	electron transport chain ($NaN \times 10^{-Inf}$)
	photosynthesis, light reaction ($NaN \times 10^{-Inf}$)
	membrane organization ($NaN \times 10^{-Inf}$)
	plastid organization ($NaN \times 10^{-Inf}$)
	protein targeting ($NaN \times 10^{-Inf}$)
	cellular protein localization ($NaN \times 10^{-Inf}$)
	protein transport ($NaN \times 10^{-Inf}$)
	macromolecule localization ($NaN \times 10^{-Inf}$)
	intracellular transport ($NaN \times 10^{-Inf}$)
	signal recognition particle, endoplasmic reticulum targeting (1.06×10^{-2})
12	
13	6-phosphogluconolactonase activity (1.96×10^{-2})
	omega-3 fatty acid desaturase activity (3.92×10^{-2})
	water homeostasis (3.97×10^{-2})
	cellular potassium ion homeostasis (4.96×10^{-2})
14	protein heterodimerization activity (2.35×10^{-3})

	autophagic vacuole (1.94×10^{-2})
	detection of fungus (2.43×10^{-2})
15	cell-cell signaling (1.70×10^{-2})
	signal transducer activity (3.65×10^{-2})
16	jasmonic acid biosynthetic process (6.77×10^{-3})
	oxylipin metabolic process (8.6×10^{-3})
	immune response (1.64×10^{-2})
	allene-oxide cyclase activity (3.32×10^{-2})
	cytokinin dehydrogenase activity (4.98×10^{-2})
17	transcription factor activity (3.15×10^{-2})
18	response to chitin (2.17×10^{-4})
	response to organic substance (2.95×10^{-3})
	defense response to virus (7.87×10^{-3})
	response to wounding (1.68×10^{-2})
	NAD+ ADP-ribosyltransferase activity (4.88×10^{-2})
19	sphingolipid delta-4 desaturase activity (1.53×10^{-2})
20	cellular response to sucrose starvation (4.05×10^{-3})
	aromatic amino acid transmembrane transporter activity (6.64×10^{-3})
	asparagine synthase (glutamine-hydrolyzing) activity (9.96×10^{-3})
	sinapate 1-glucosyltransferase activity (1.33×10^{-2})
	NAD+ ADP-ribosyltransferase activity (1.99×10^{-2})
	fatty acid (omega-1)-hydroxylase activity (2.32×10^{-2})
	neutral amino acid transmembrane transporter activity (2.65×10^{-2})
	response to fructose stimulus (4.45×10^{-2})
	pyruvate kinase activity (4.64×10^{-2})
	response to absence of light (4.85×10^{-2})
21	zinc ion binding (1.21×10^{-3})
	cation binding (1.31×10^{-2})
	metal ion binding (2.07×10^{-2})
22	prephenate dehydratase activity (8.15×10^{-3})
	arogenate dehydratase activity (8.15×10^{-3})
	beta-amylase activity (1.22×10^{-2})
	response to wounding (3×10^{-2})
	L-phenylalanine biosynthetic process (4.64×10^{-2})
23	response to wounding (2.34×10^{-7})
	response to jasmonic acid stimulus (1.56×10^{-4})
	jasmonate O-methyltransferase activity (1.05×10^{-2})
	jasmonic acid biosynthetic process (1.46×10^{-2})
	oxylipin metabolic process (1.85×10^{-2})
	defense response (2.18×10^{-2})
	jasmonic acid mediated signaling pathway (2.45×10^{-2})
	1,2-diacylglycerol 3-beta-galactosyltransferase activity (3.15×10^{-2})
24	response to water deprivation (3.18×10^{-6})
	protein serine/threonine phosphatase complex (1.97×10^{-5})
	response to endogenous stimulus (1.75×10^{-4})
	negative regulation of abscisic acid mediated signaling (3.42×10^{-4})

	negative regulation of cell communication (4.97×10^{-4})
	protein serine/threonine phosphatase activity (8.45×10^{-4})
	protein amino acid dephosphorylation (1.90×10^{-3})
	phosphatase activity (9.75×10^{-3})
	response to abiotic stimulus (1.11×10^{-2})
25	trans-zeatin O-beta-D-glucosyltransferase activity (1.01×10^{-3})
	cis-zeatin O-beta-D-glucosyltransferase activity (1.01×10^{-3})
	glucuronosyltransferase activity (1.41×10^{-3})
	UDP-glycosyltransferase activity (1.75×10^{-2})
	glucosyltransferase activity (2.05×10^{-2})
	transferase activity (3.09×10^{-2})
26	glutamate dehydrogenase activity (2.27×10^{-3})
	glutamate dehydrogenase [NAD(P)+] activity (4.53×10^{-3})
	nitrate reductase activity (4.53×10^{-3})
	nitric oxide biosynthetic process (1.64×10^{-2})
	ER body (1.72×10^{-2})
	nitrate assimilation (3.28×10^{-2})
27	secondary metabolic process (1.54×10^{-3})
28	
29	acyl-[acyl-carrier-protein] hydrolase activity (1.99×10^{-2})
	bis(5'-adenosyl)-pentaphosphatase activity (2.99×10^{-2})
	cell plate formation involved in plant-type cell wall biogenesis (4.1×10^{-2})
	para-aminobenzoic acid metabolic process (4.1×10^{-2})
